# Effect of humic substances on the fraction of heavy metal and microbial response

**DOI:** 10.1038/s41598-024-61575-5

**Published:** 2024-05-16

**Authors:** Mengmeng Wang, Gangfu Song, Zhihong Zheng, Zhixin Song, Xiao Mi, Jiajun Hua, Zihang Wang

**Affiliations:** North China University of Water Resources and Electric Power, Zhengzhou, 450046 People’s Republic of China

**Keywords:** Soil, Molybdenum, Fulvic acid and humic acid, BCR, Bacterial, Ecology, Environmental sciences

## Abstract

Contamination of soils by Molybdenum (Mo) has raised increasing concern worldwide. Both fulvic acid (FA) and humic acid (HA) possess numerous positive properties, such as large specific surface areas and microporous structure that facilitates the immobilization of the heavy metal in soils. Despite these characteristics, there have been few studies on the microbiology effects of FA and HA. Therefore, this study aimed to assess the Mo immobilization effects of FA and HA, as well as the associated changes in microbial community in Mo-contaminated soils (with application rates of 0%, 0.5% and 1.0%). The result of the incubation demonstrated a decrease in soil pH (from 8.23 ~ 8.94 to 8.05 ~ 8.77). Importantly, both FA and HA reduced the exchangeable fraction and reducible fraction of Mo in the soil, thereby transforming Mo into a more stable form. Furthermore, the application of FA and HA led to an increase in the relative abundance of Actinobacteriota and Firmicutes, resulting in alterations to the microbial community structure. However, it is worth noting that due to the differing structures and properties of FA and HA, these outcomes were not entirely consistent. In summary, the aging of FA and HA in soil enhanced their capacity to immobilization Mo as a soil amendment. This suggests that they have the potential to serve as effective amendments for the remediation of Mo-contaminated soils.

## Introduction

Environmental pollution caused by heavy metals (HMs) poses a significant challenge to our planet. The challenge lies in the persistence of heavy metals in the environment. Unlike organic compounds that can break down over time, heavy metals do not undergo natural decomposition processes. As a result, they accumulate in soil, water bodies, and organisms through the food chain^1^. This accumulation can have severe consequences for both the ecosystem and human health. Furthermore, the management of HM pollution is a complex task. Therefore, finding sustainable and efficient solutions to tackle HM pollution is crucial.

The sources of heavy metals in soil primarily come from natural and anthropogenic activities. These include atmospheric deposition, mining, excessive fertilizer application, and wastewater irrigation^1–3^. Heavy metal molybdenum (Mo) is widely used in metallurgy and the chemical industry, making it an essential mineral resource^4^. Due to their resistance to natural decomposition, heavy metals persist in the soil. Previous studies indicate that Mo is a vital element for living organisms, playing a significant role in enzymes such as xanthine oxidase and sulfite oxidase^5,6^. However, high concentrations of Mo can be detrimental to both organisms and their surroundings. For example, in somerset, elevated levels of Mo in grass can lead to dirty ruminants, decreased milk production, and, in severe cases, even death among cows^7^. Furthermore, excessive ingestion of Mo can cause health issues such as diarrhea, anemia, growth retardation and lung etc. diseases^8,9^. In certain regions, the Mo content in soil exceeds the background value^4,10^. Therefore, it is necessary to implements measures to control heavy metal pollution in soil. In the past, most scholarly attention has been directed towards the removal of Mo from water^11–13^, with limited focus on its removal from soil^14^.

Generally speaking, the management of heavy metal pollution has comprised a mix of regulatory measures, remediation techniques, and pollution prevention strategies. Such as, setting limits for heavy metal emissions and concentrations in soil, water, and air. But Regulatory measures often lag behind the latest scientific findings and may not be stringent enough to protect environmental and human health. The reported repair technologies include physical remediation technologies, chemical remediation technologies and combined remediation technologies, etc^15^. However, high cost, soil degradation, equipment abandonment and secondary pollution are characteristics of some remediation technologies^16–19^. Pollution prevention strategies include clean production technologies and product substitution, but these strategies require significant investment and may not be feasible for all industries. And there is also a reliance on industry compliance and the development of economically viable alternatives. However, due to their potential adverse effects on the environment, researchers are seeking new amendments for the remediate heavy metal-contaminated soil.

Humic substances (HS) are ubiquitous in soil and water and play vital roles in the environment. HS is prominent components of soil organic matter, containing various functional groups such as carboxyl and hydroxyl^20^. The main constituents of HS include fulvic acid (FA) and humic acid (HA), which are natural components of soil and pose no harm to the soil environment. HA and FA are considered promising for several applications due to their unique properties and benefits. HA and FA can improve soil structure, enhance water retention, and increase nutrient availability to plants. This is crucial for agricultural productivity and sustainability^21^. They can help to immobilize harmful substances, reducing their bioavailability and toxicity in the environment^22,23^. In water treatment, HA and FA can act as natural chelators, binding to potentially toxic metals and facilitating their removal from water supplies. Their structure allows them to form complexes with contaminants, which can then be filtered out^24^. As stable organic compounds, humic substances are significant in the context of carbon sequestration. They can help mitigate climate change by retaining carbon in soil for extended periods, reducing the amount released into the atmosphere as carbon dioxide^25^. The promise of HA and FA lies in their versatility, natural origin, and the relative sustainability of their applications. Continued research into these substances is expanding their potential uses and improving our understanding of their benefits in both environmental and economic contexts.

Microorganisms play a crucial role in soil, and high-throughput sequencing technology has facilitated research on microorganisms^1^. Microbial richness and diversity can, to some extent, reflect the quality and status of soil^26^. Heavy metals have the potential to alter the community structure of microorganisms, thereby affecting their richness and diversity^27^. Furthermore, heavy metals can significantly inhibit soil microbial activity^27–30^. For instance, Golebiewski discovered that Zn decreases microbial diversity and richness in soil^31^. In contrast, some studies have reported no significant relationship between heavy metals and microbial communities^29,30^. Therefore, the relationship between heavy metals and microbial communities is complex. Currently, it is unclear how the application of FA and HA affects the microbial community structure in Mo-contaminated soil.

To comprehensively understand the long-term effects of FA and HA on Mo-polluted soils and their impacts on microorganisms, it is necessary to conduct systematic investigations. In this study, we examined the influence of humic substances (FA and HA) at different concentrations (0%, 0.1%, 0.5%, 1%) and under varying aging periods (1, 2, 5, 9, and 13 weeks) in soils contaminated with heavy metal Mo. The objectives of this research were as follows: (1) to investigate the impact of FA and HA on the bioavailability of Mo in polluted soil, (2) to assess their effects on microbial diversity indices and community structure, and (3) to explore the relationships between changes in Mo bioavailability, soil properties, and bacterial composition in polluted soils. The findings from this study could serve as a theoretical foundation for understanding the migration and transformation of heavy metal Mo in polluted soil.

## Materials and methods

### Soil sample

The clean soil selected for this study was collected from North China University of Water Resources and Electric Power (113° 47.661′ E, 34° 46.789′ S) (pH = 8.49, loess). The soil was air-dried and sieved through a 2 mm mesh to remove stones, plant roots, and other debris. The FA and HA were provided by Shanghai Yuanye Bio-Technology Co., Ltd (Shanghai, China).

To prepare the required treatment, the quantity of exogenous heavy metals (Na_2_MoO_4_·2H_2_O) needed was calculated, and a solution was prepared. This solution was then added to the soil through spraying to achieve a Mo concentration of 300 mg/kg. The soil was thoroughly mixed and allowed to stand in a cool place for one month before use.

### Experimental designs

The dry mass of each soil treatment was 300 g. Three application rates of FA/HA were selected: 0.1%, 0.5%, and 1% (w/w)^1^. The FA/HA was then thoroughly mixed with the soil and homogenized, resulting in the following treatments: SFA0.1, SFA0.5, SFA1, SHA0.1, SHA0.5, and SHA1. A control treatment, SCK, was also prepared by spraying the same amount of distilled water onto the polluted soils. Each treatment was replicated independently three times. After thorough mixing and three days of soil stabilization, the soil was placed in an incubator for constant temperature cultivation. Irrigation with distilled water was performed every 7 days, maintaining the soil at 60% of its water-holding capacity.

Soil samples from each treatment were collected at different aging periods (1, 2, 5, 9, and 13 weeks). Each treatment's soil was air-dried and passed through a 100 mesh sieve for speciation analysis, as well as a 20 mesh sieve for pH analysis. Additionally, at the 13-week mark, the collected treatment soil was stored in a – 80 ℃ refrigerator for subsequent 16S rRNA testing.

### Analytical methods

The soil pH was measured using a pH meter (PXSJ-216F, Shanghai INESA Scientific Instrument CO., Ltd, China) at a soil-to-water ratio of 1:2.5^32^.

For the analysis of microbial community in the soil, the 16S rRNA gene was amplified using the primer sets 338F (ACTCCTACGGGAGGCAGCAG) and 806R (GGACTACHVGGGTWTCTAAT). Illumina MiSeq sequencing was employed for the analysis. For specific details, please refer to the supplementary materials.

To extract the fractions of heavy metals, the modified European Community Bureau of Reference (BCR) sequential extraction method was utilized^33–35^. This method includes the following fractions: exchangeable fraction (F1), which accounts for water-extractable, exchangeable, and carbonate-bound metals; reducible fraction (F2), consisting of metals bound to Fe–Mn oxides; oxidizable fraction (F3), accounting for metals bound to organic matter and sulphides; and residual fraction (F4), consisting of metals bound to mineral lattices^36^. For specific details, please refer to the supplementary materials.

The soil samples were digested in a microwave unit (ETHOS UP, Milestone, Italy) using a mixture of HCI/HNO3/HF, following the methodology established in previous studies^37,38^. The content of Mo was determined using an atomic absorption spectrophotometer (TAS-990, Beijing Purkinje General Instrument Co., Ltd, China).

### Statistical analysis

This study ensured quality assurance and control by employing duplicate samples, standard reference samples, and control treatments. The recoveries of both the chemical fractions of Mo and the total amount of Mo were within the range of 90–105%. All tests were conducted in triplicate, resulting in a standard deviation of less than 5%, and the subsequent results were averaged.

For graphical representation, all graphs in this study were created using Origin 2023b.

## Results

### The effect of HA and FA dosage on soil pH

**Figure 1:**
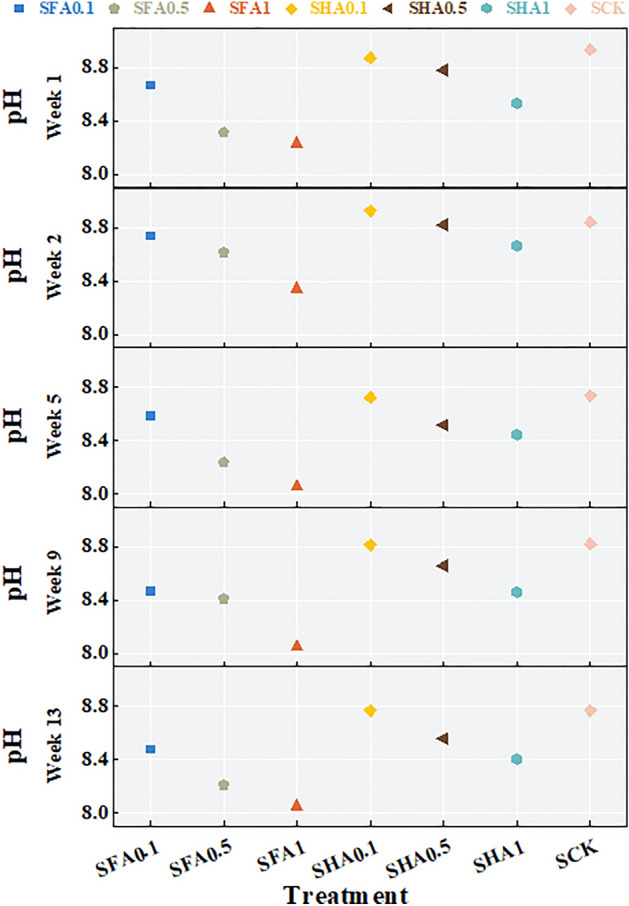
Variation trends of pH in soil under different treatments with time.

The addition of FA and HA resulted in decreased pH values, as depicted in Fig. 1 and Table 1.

**Table 1:** pH changes during the test period.

	Week 1	Week 2	Week 5	Week 9	Week 13
SFA0.1	8.67	8.74	8.59	8.46	8.48
SFA0.5	8.31	8.62	8.24	8.41	8.21
SFA1	8.23	8.35	8.06	8.05	8.05
SHA0.1	8.87	8.93	8.73	8.82	8.77
SHA0.5	8.78	8.82	8.51	8.66	8.56
SHA1	8.53	8.67	8.44	8.46	8.40
SCK	8.94	8.84	8.74	8.82	8.77

As the proportion of FA addition increased, the pH of each treatment after weekly soil cultivation showed a declining trend, mirroring the trend observed in HA. In SFA0.1, the overall pH initially increased and then decreased, reaching its peak value in week 2 (pH = 8.74), followed by a decrease below the pH value of week 1 in week 13 (week 1: 8.67, week 13: 8.48). The data trends in the other processing groups were largely similar to SFA0.1, with the pH data of week 13 being lower than that of week 1, except for the maximum pH value of SCK, which appeared in week 1. The pH values in the 13th week of each treatment decreased by 0.10 (SHA0.1) to 0.23 (SHA0.5) compared to the first week, respectively.

Comparison of the addition of FA and HA in the same proportion revealed that the pH value of SHA after soil cultivation was higher than that of the SFA treatment. With the exception of week 2, where the maximum value of each treatment appeared at SHA0.1 (pH = 8.93), the pH values of the other 4-week treatments at SCK were not less than those of SFA and SHA.

### The effect of HA and Fa dosage on the speciation and chemical forms of molybdenum

The total amount of heavy metals can to some extent indicate the source of pollution and potential pollution. However, it cannot reflect the bioavailability and ecological toxicity of heavy metals, which are determined by their occurrence forms in soils^39^.

**Figure 2:**
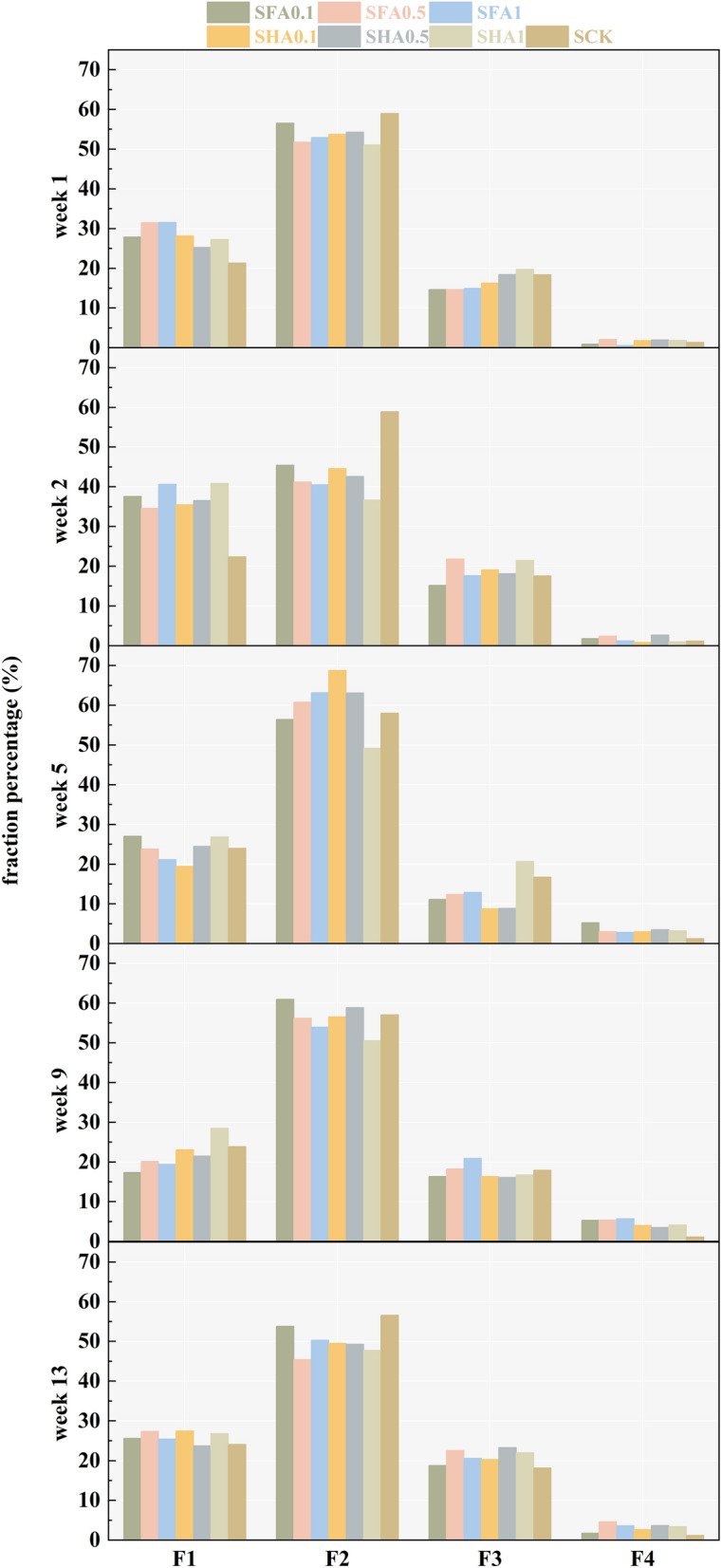
Variation trends of Mo forms in soil under different treatments with time.

Changes in the distribution of Mo forms over time are presented in Fig. 2. Overall, the proportion of F1 ranges from 17.38 to 40.88% (average: 26.92%), F2 ranges from 36.69 to 68.77% (average: 52.54%), F3 ranges from 8.78 to 23.29% (average: 17.12%), and F4 ranges from 0.57 to 5.73% (average: 2.69%). F2 is the dominant form.

In the SFA treatments, after 13 weeks of cultivation, there is a general decrease in the proportion of F1 (SFA0.1: 27.88–25.60%, SFA0.5: 31.50–27.37%, SFA1: 31.56–25.43%). The F1 values at week 13 are lower than those at week 1 for all treatments. The trend of F2 is similar to that of F1 (from 56.53%, 51.74%, 52.91% to 53.83%, 45.43%, 50.31%). On the other hand, the proportions of F3 and F4 show an opposite trend, with an overall increase reaching 18.80%, 22.58%, and 20.61%, respectively. Therefore, it can be concluded that adding FA to the soil promotes the transformation of F1 and F2 towards F3 and F4, effectively reducing the migration of Mo in the soil.

In the SHA treatments, after 13 weeks of cultivation, the trend of F1 is not significant. The proportion of F2 shows a general decrease, while the proportions of F3 and F4 show an overall increase. Adding different proportions of HA to the soil promotes the transformation from F2 to F3 and F4. Adding HA can effectively reduce the migration of Mo in the soil.

### The changes of bacterial community under different treatments

**Figure 3:**
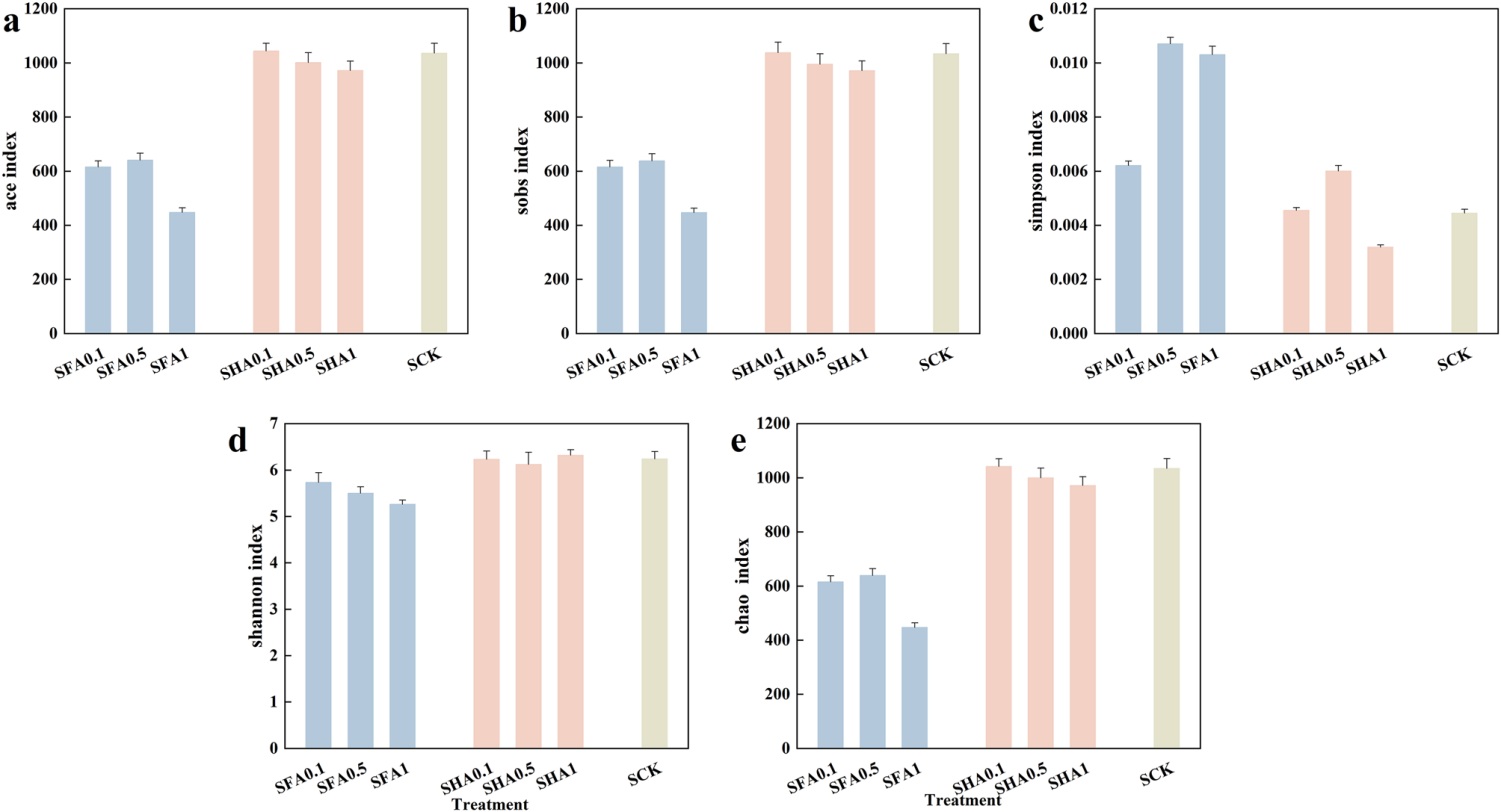
(**a**) Ace index, (**b**) sobs index, (**c**) Simpson index, (**d**) Shannon index, (**e**) chao index, error bars represent ± SE of triplicates (n = 3).

Figure 3 displays the alpha diversity indices of bacterial 16S rRNA gene in each treatment, including ace index, sobs index, simpson index, shannon index, and chao index. The results show that in the FA treatments, ace index, sobs index, shannon index, and chao index were lower compared to the control and SHA treatments. These findings suggest that the presence of SFA can reduce the richness and diversity of species. Conversely, there is only a slight difference in these indices between the soil with added HA and the control.

**Figure 4:**
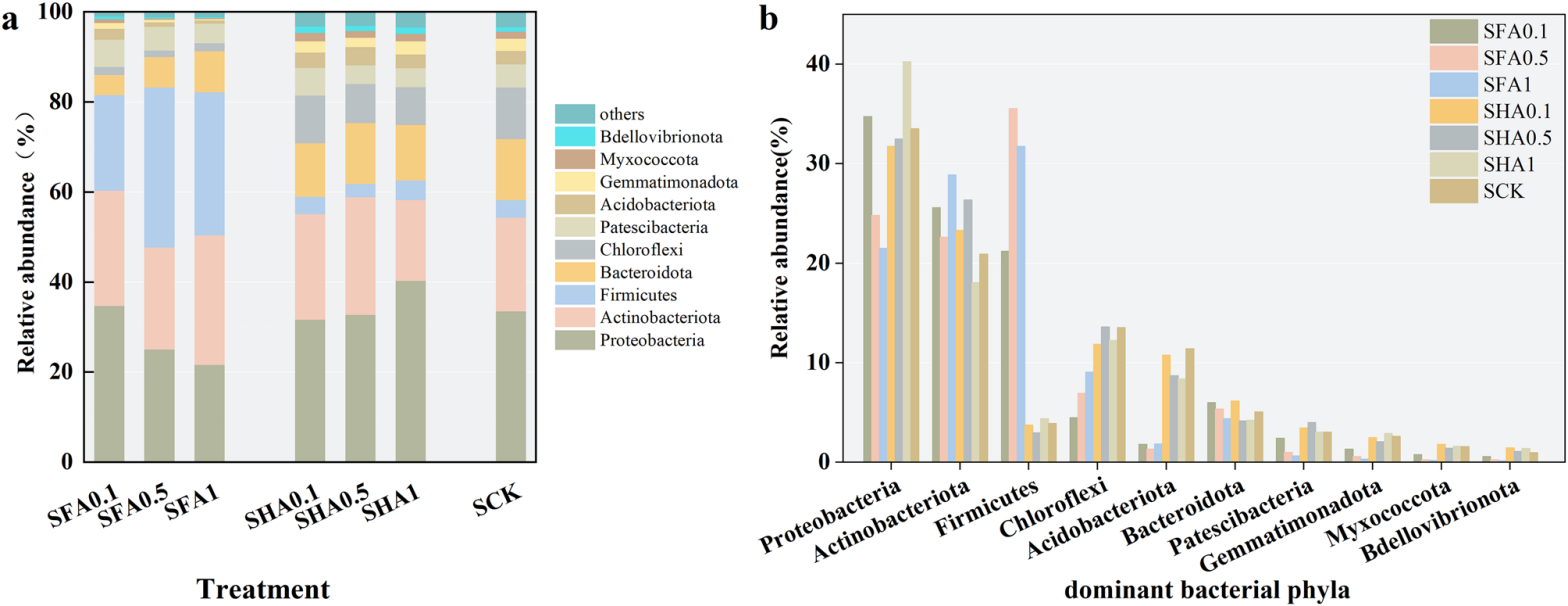
Soil bacterial community (**a**) predominant phyla, (**b**) the relative abundances of different phyla from different treatments.

The analysis of bacterial community revealed that the application of FA and HA altered the structure of the bacteria community. Figure 4a,b present the relative abundance of the week13 soil bacterial community in the different treatments. Regardless of FA and HA levels, we observed ten dominant bacterial phyla in the soil samples: Proteobacteria (21.63–40.32%), Actinobacteriota (17.90–28.81%), Firmicutes (2.92–35.55%), Chloroflexi (4.47–13.51%), Acidobacteriota (1.37–11.48%), Bacteroidota (4.15–6.12%), Patescibacteria (0.67–4.02%), Gemmatimonadota (0.32–2.90%), Myxococcota (0.22–1.82%), and Bdellovibrionota (0.17–1.45%).

In comparison to the control, the relative abundances of Actinobacteriota and Firmicutes were higher (25.59%, 22.62%, and 28.81% versus 20.79%) and (21.23%, 35.55%, and 31.74% versus 3.95%) in the SFA0.1, SFA0.5, and SFA1 treatments. The relative abundance of Patescibacteria and Bdellovibrionota also increased (3.43%, 4.02%, and 3.04% versus 3.04%) and (1.45%, 1.12%, and 1.42% versus 0.99%) in the SHA0.1, SHA0.5, and SHA1 treatments. Conversely, the addition of FA and HA resulted in decreased abundances of Chloroflexi and Acidobacteriota (4.47%, 6.81%, 9.10%, 11.93%, 13.50%, and 12.32% in SFA0.1, SFA0.5, SFA1, SHA0.1, SHA0.5, and SHA1 treatments versus 13.51% in the control; 1.81%, 1.37%, 1.82%, 10.60%, 8.72%, and 8.41% in SFA0.1, SFA0.5, SFA1, SHA0.1, SHA0.5, and SHA1 treatments versus 11.48% in the control) in the soil. The abundances of Patescibacteria, Gemmatimonadota, Myxococcota, and Bdellovibrionota decreased as the FA application rate increased from 0.1 to 1%. By comparing the effects of adding FA and HA on soil microorganisms, it can be observed that the impact of FA and HA addition on microorganisms differs.

**Figure 5:**
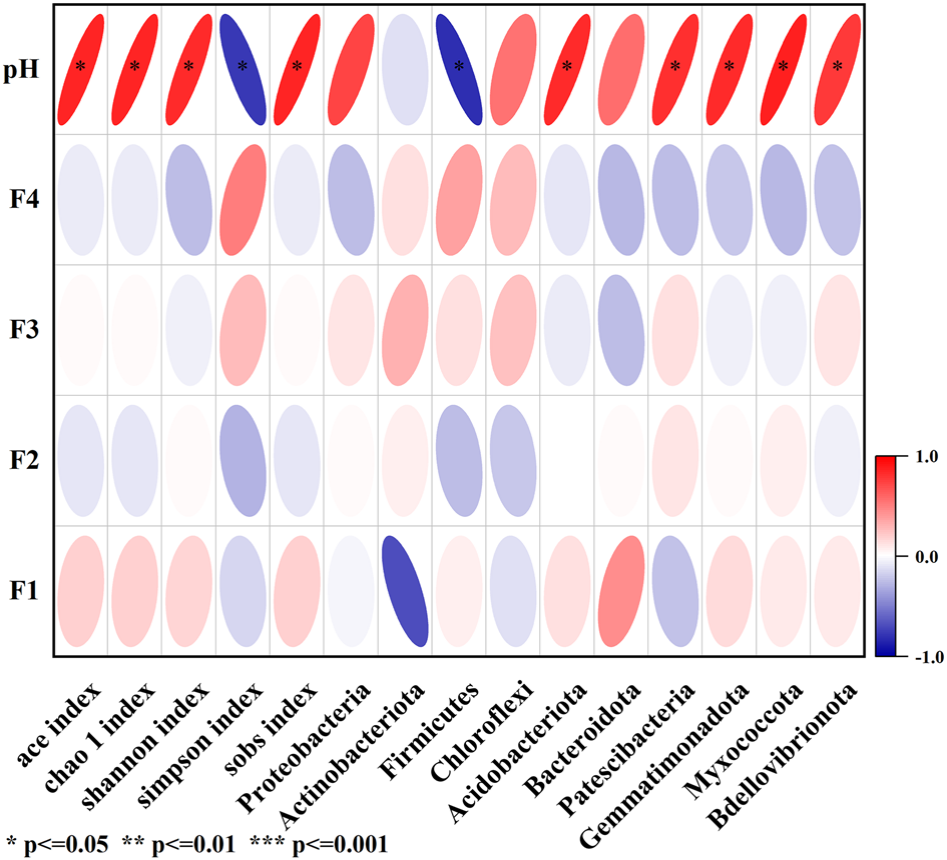
Relationship of bacterial community and soil properties (*p ≤ 0.05, **p ≤ 0.01, ***p ≤ 0.001).

In this study, significant relationships were observed between pH and the six predominant bacterial phyla (p < 0.05). Specifically, pH was positively correlated with the relative abundance of Acidobacteriota, Patescibacteria, Gemmatimonadota, Myxococcota, and Bdellovibrionota, but negatively correlated with that of Firmicutes (Fig. 5).

## Discussion

In this study, both FA and HA were found to have an impact on the morphology of soil Mo. However, their effects and intensities varied, which could be attributed to the structural and property characteristics of FA and HA. Humic acids, in general, can influence the form and activity of heavy metals through various complex mechanisms. For example, they can form organic–inorganic complexes with inorganic colloidal components or complexes with structural cations in inorganic components, thereby altering the surface properties and adsorption capacity of soil^40,41^. Additionally, they can also change the form of heavy metal ions themselves through chelation^42^.

The addition of FA and HA during the experiment resulted in a varying degree of decrease in soil pH. Soil pH can affect the bioavailability of Mo from multiple perspectives. However, changes in soil pH represent only one aspect of how FA and HA influence soil properties. Moreover, FA and HA can enhance the structure of soil microbial communities. Microorganisms play a crucial role in the migration and transformation of Mo through processes such as oxidation/reduction, adsorption/desorption, precipitation/dissolution, thus affecting its bioavailability.

Studies have demonstrated that low molecular weight FA can enhance the effectiveness of heavy metals, whereas HA exhibits an inhibitory effect. The results reveal that FA promotes the increase in effective Cd content, while HA has a certain inhibitory and passivation effect on the effective extraction of Cd and Pb^43^. The findings of this study indicate that both FA and HA reduced the bioavailability of Mo. In fact, both FA and HA facilitated the transformation of soil Mo from F1 and F2 to F3 and F4, leading to a decrease in the bioavailability of Mo. It is important to note that factors influencing the bioavailability of heavy metals are not limited to FA and HA alone, but may also involve other factors. Therefore, further research is necessary.

In this study, Proteobacteria was identified as one of the most abundant phyla in the soil under investigation. Proteobacterium is a dominant bacterial phylum found in environments with high levels of heavy metal pollution, such as mine sediments, surface water, and heavy metal-contaminated soil. It is widely distributed and prevalent^44–46^. The farmland area soil also contains a significant amount of Proteobacteria and Actinobacteria. These beneficial bacteria may exhibit reduced sensitivity to heavy metals due to their tolerance gene group^47^. As a result, Proteobacteria and Actinobacteria can coexist in extreme environments and are considered capable of remediating heavy metal pollution^48^. After the addition of soil amendments, Proteobacteria, Actinobacteria, Acidobacteria, and Chloroflexi also became dominant bacterial phyla^1,49^. However, these dominant communities are not exactly the same, which may be attributed to the complex and diverse bacterial communities in different mining areas and habitats. It may be necessary to consider environmental variables in order to have a more comprehensive discussion.

Furthermore, environmental variables are believed to play a crucial role in regulating the adaptation of microbes to different levels of heavy metal contamination^50^. pH is a significant factor that affects the structure of the microbial community^32,51^. Over the past decade, numerous studies have confirmed the main role of pH in bacterial community diversity and composition^32,52,53^. For instance, pH was found to be positively correlated with the relative abundance of d-Proteobacteria and Bacteroidetes^52^.

## Conclusions

The incubation experiment revealed that the application of FA and HA led to improvements in the properties of Mo-contaminated agricultural soils. Moreover, the application of FA and HA resulted in reduced bioavailability of Mo in the soil, thereby promoting the percentages of F3 and F4 while reducing the component percentages of F1 and F2. The application of FA and HA also brought about changes in the diversity and structure of the bacterial community, particularly the phyla Firmicutes, Acidobacteriota, Patescibacteria, Gemmatimonadota, Myxococcota, and Bdel-lovibrionota, which were driven by variations in soil pH. These changes, in turn, altered the bioavailability of heavy metals in the soil. Based on these findings, it can be concluded that the long-term effect of FA and HA on the stabilization of Mo in agricultural soil makes it a potential amendment for stabilizing Mo in contaminated agricultural soils.

### Supplementary Information

Supplementary Information.
