# Supplementary material for: Effect of humic substances on the fraction of heavy metal and microbial response
Source: Sci Rep. 2024 May 16;14:11206. doi: 10.1038/s41598-024-61575-5 (PMC11099172; doi:10.1038/s41598-024-61575-5)
Supplement: Supplementary file 1 — Supplementary Information. [file 41598_2024_61575_MOESM1_ESM.pdf]

Supporting information for

Effect of humic substances on the fraction of heavy metal and microbial response

Mengmeng Wang, Gangfu Song*, Zhihong Zheng, Zhixin Song, Xiao Mi*, Jiajun Hua, Zihang Wang

North China University of Water Resources and Electric Power，Zhengzhou 450046, PR China

Method for microbial community

For the analysis of microbial community in the soil, the 16S rRNA gene was amplified using the primer sets 338F (ACTCCTACGGGAGGCAGCAG) and 806R (GGAC-TACHVGGGTWTCTAAT).

The experiment used TransGen AP221-02: TransStart Fastpfu DNA Polymerase, 20 μl Reaction system:

5×FastPfu Buffer	4 μl
2.5 mM dNTPs	2 μl
Forward Primer (5 μM)	0.8 μl
Reverse Primer (5 μM)	0.8 μl
FastPfu Polymerase	0.4 μl
BSA	0.2 μl
Template DNA	4 ng
Add dd H_2O to	20μl

PCR reaction parameters:

a. 1× (3 minutes at 95°C)

b. Number of cycles × (30 seconds at 95 ℃; 30 seconds at annealing temperature℃; 45 seconds at 72℃)

c. 10 minutes at 72℃, 10° C until halted by user

Modified European Community Bureau of Reference (BCR) sequential extraction scheme[1]

Step 1 (exchangeable fraction, F1): 20 mL of 0.11 mol·L−1 HOAc was added to 0.5 g of sample in a centrifuge tube and shaken for 16 h at room temperature. The extract was separated from the solid phase by centrifugation at 3000 rpm for 20 min.

The supernatant liquid was decanted into a polypropylene centrifuge tube and stored at 4 °C in a refrigerator.

Step 2 (reducible fraction, F2): 20 mL of 0.1 mol·L−1 hydroxylamine hydrochloride was added to the residue from Step 1. The rest of the procedure follows that of Step 1.

Step 3 (oxidizable fraction, F3): 5 mL of 8.8 mol·L−1 hydrogen peroxide was added to the residue from Step 2 for 1 h, and the tube was heated until the volume was reduced to about 2–3 mL. Another 5 mL of 8.8 mol·L−1 hydrogen peroxide was added and heating continued until the volume was almost reduced to dryness. After cooling, 20 mL of 1.0 mol·L−1 ammonium acetate was added to the residue. The remainder of the procedure is the same as that of Step 1.

Step 4 (residual fraction, F4): after drying, the residue from Step 3 was digested in a mixture of HNO3, HCl, and HF.

References

1 Ure, A. M., Quevauviller, P., Muntau, H. & Griepink, B. Speciation of Heavy Metals in Soils and Sediments. An Account of the Improvement and Harmonization of Extraction Techniques Undertaken Under the Auspices of the BCR of the Commission of the European Communities. *International Journal of Environmental Analytical Chemistry* **51**, 135-151, doi:https://doi.org/10.1080/03067319308027619 (1993).
